# A drink equals how many cigarettes? Equating mortality risks from alcohol and tobacco use in Canada

**DOI:** 10.3389/fpubh.2024.1331190

**Published:** 2024-02-27

**Authors:** Harpreet Jaswal, Ivneet Sohi, Jürgen Rehm, Samuel Churchill, Adam Sherk, Tim Stockwell, Christine Levesque, Nitika Sanger, Hanie Edalati, Peter R. Butt, Catherine Paradis, Kevin D. Shield

**Affiliations:** ^1^Institute for Mental Health Policy Research, Centre for Addiction and Mental Health, Toronto, ON, Canada; ^2^Dalla Lana School of Public Health, University of Toronto, Toronto, ON, Canada; ^3^Campbell Family Mental Health Research Institute, Centre for Addiction and Mental Health, Toronto, ON, Canada; ^4^Canadian Institute for Substance Use Research, University of Victoria, Victoria, BC, Canada; ^5^College of Medicine, University of Saskatchewan, Saskatoon, SK, Canada; ^6^Canadian Centre on Substance Use and Addiction, Ottawa, ON, Canada

**Keywords:** alcohol use, tobacco use, Canada, mortality metrics, guidance on alcohol and health

## Abstract

**Objective:**

To quantify and communicate risk equivalencies for alcohol-and tobacco-attributable mortality by comparing per standard drinks consumed to per number of cigarettes smoked in Canada.

**Methods:**

Alcohol-and tobacco-attributable premature deaths (≤75 years of age) and years of life lost (YLL) were estimated using a lifetime risk modeling approach. Alcohol-attributable death statistics were obtained from the 2023 Canadian Guidance on Alcohol and Health data source. Tobacco-attributable death statistics were derived from the Mortality Population Risk Tool (MPoRT) model.

**Results:**

The risk of alcohol use on premature death and YLL increased non-linearly with the number of drinks consumed, while the risk for tobacco use on these two measures increased linearly with the number of cigarettes smoked. Males who consumed 5 drinks/day—a standard drink contains 13.45 grams of alcohol in Canada—had an equivalent risk as smoking 4.9 cigarettes/day (when modeling for premature death) and 5.1 cigarettes/day (when modeling for YLL). Females who consumed 5 drinks/day experienced an equivalent risk as smoking 4.2 cigarettes/day for premature deaths and YLL. At all levels of alcohol consumption females and males who consumed <5 drinks/day have less risks from consuming a standard drink than from smoking a cigarette. For males who consumed 5 drinks/day, the increased risks of death from per drink consumed and per cigarette smoked were equal.

**Conclusion:**

Risk equivalencies comparing alcohol use to tobacco use could help people who drink improve their knowledge and understanding of the mortality risks associated with increased number of drinks consumed per day.

## Introduction

Alcohol use is a causal risk factor for over 230 disease conditions based on the three-digit International Classification of Diseases, Revision 10 (ICD-10) codes (1). These health risks, however, are not always well understood by the public for a multitude of reasons. Studies have shown that people generally have difficulties understanding, evaluating, and communicating mortality risk associated with alcohol use (2). Although the general public tends to be aware of the protective effects of alcohol use on ischemic heart disease, ischemic stroke, and diabetes, especially for people who drink and who drink low amounts or do not engage in heavy episodic drinking (1), they, for the most part, are unaware of the many of the diseases that excessive alcohol consumption can cause (3, 4). For example, alcohol use is a leading cause of many forms of cancer and associated medical complications (1). Gaps in this knowledge is a major reason for why people often underestimate the risks of excessive drinking, and the catastrophic drinking patterns that often result. Providing information on the risk of alcohol use has been found to improve health literacy, decrease peoples’ intention to drink, and decrease the harms associated with alcohol consumption (5). Unfortunately, communicating this information about risks has not always been conducted effectively. As a prevention strategy, it is often underutilized by health and public health professionals.

People’s understanding of health risks related to alcohol use can be improved by communicating risk equivalencies. A ‘risk equivalency’ refers to a comparative assessment whereby different risks of health behaviors or related conditions are compared or evaluated so they can be expressed in terms of a common unit or metric — for an example, please see reference 6. Providing risk equivalency information can aid people in improving their understanding of the risks or health impacts they may experience acutely or over time from excessive alcohol use. Such information can also help individuals assess their situation(s) and prioritize their responses to alcohol availability. In contrast to alcohol use, the risk of tobacco use are well known to the general public (7–9). Various studies from different countries have shown that people are generally knowledgeable about diseases related to tobacco use and second-hand smoke exposure (10, 11). Because of this greater awareness about tobacco’s risks for harm, risk equivalencies can add value when communicating about risks associated with alcohol use — i.e., equating health loss or harm from standard drinks to health loss or harm from the number of cigarettes smoked. The present study uses Canadian data to address this gap in public health communication, by quantifying the risk equivalencies for alcohol and tobacco use. Premature death and years life lost (YLL) are the two main measures used to generate these equivalencies.

## Methods

The lifetime risks of an alcohol- or tobacco-attributable death were operationalized using the risk of a premature death (i.e., a death which occurred among people 75 years of age and younger) and YLL. YLL were estimated based on the age of death and sex-specific lifetables for 2019, as obtained from Statistics Canada (12). This data source contained lifetables of probabilities on life expectancy and mortality for Canadians by age and sex.

### Defining exposure and reference groups

Exposures to alcohol and tobacco were operationalized using different dimensions. For the present study alcohol use was operationalized as standard drinks consumed per week (in the analyses, this was converted to per day). In Canada, a standard drink comprises 13.45 grams of ethanol (13). Exposure to tobacco was operationalized as the average number of cigarettes smoked per day. To estimate alcohol-and tobacco-attributable deaths, and YLL a theoretical minimum risk exposure level (TMREL) for lifetime abstention was applied. No assumptions were made about the level of exposure for alcohol use that would result in the lowest risk of overall health loss or harm.

### Lifetime risk of an alcohol-attributable death

The lifetime risk estimates of an alcohol-attributable death and YLL were extracted using statistics from the 2023 Canadian Guidance on Alcohol and Health (see (14)). These 2023 statistics were used to generate alcohol use risks for people who consumed 0 to 5 drinks per day using a lifetime risk approach containing multiple steps and data sources (14, 15).

The first step in this approach was to estimate the number of cause-, sex- and age-specific alcohol-attributable deaths in Canada for 2019; this was carried out using a Levin-based population-attributable fraction method that combined data on alcohol exposure, relative risk estimates and mortality estimates (16, 17). The data on alcohol exposure were obtained from the Canadian Alcohol and Drug Use Monitoring Survey (CADUMS); the Canadian Tobacco, Alcohol and Drugs (CTADS) Survey; and Canada’s national statistical office, Statistics Canada (18). The data on the relative risk estimates were obtained from meta-analyses found in the literature (14). And the data on mortality estimates were obtained from the Statistics Canada Canadian Vital Statistics database (CVSD) (19).

The second step in the approach was to estimate the number of non-alcohol-attributable deaths in Canada (i.e., the deaths that would occur if no one person consumed alcohol); this was done by subtracting the number of alcohol-attributable deaths (estimated in the first step) from the total number of deaths. The number of non-alcohol-attributable deaths in Canada was subsequently divided by the population of Canada [via Statistics Canada (20)], yielding the risk measure for non-alcohol-attributable deaths - i.e., the risk of death among lifetime abstainers.

The third and final step in the approach was to estimate the lifetime risk of an alcohol-attributable death. To do this, the age- and sex-specific risks of an alcohol-attributable death (estimated in the second step) were summed across the life course (from age 15 years and onward). The age at which an alcohol-attributable death occurred was then used to estimate the alcohol-attributable YLL.

Because the lifetime risk of an alcohol-attributable death was characterized as a measure of public health impact, principally to provide guidance on alcohol use that is considered low risk, the modeling for the lifetime risk approach did not incorporate health loss or harm from causes such as alcohol poisoning, alcohol use disorders, or alcohol cardiomyopathy (14, 15). The latter condition, for instance, disproportionately affects people with alcohol use disorders and/or those who engage in heavy chronic drinking (21, 22). For additional information about how lifetime risks of premature death and the YLL attributable to alcohol were modeled, please see the Appendix.

### Lifetime risk of a tobacco-attributable death

The lifetime risk of a tobacco-attributable death and YLL in Canada were modeled using data from the Mortality Population Risk Tool (MPoRT) (23). The MPoRT was developed and validated using 2001 to 2008 exposure data from the Canadian Community Health Surveys and mortality data from the Canadian Registered Persons Database (23). The MPoRT estimates the risk of death based on a person’s age, sex, neighborhood deprivation, education, immigration status, smoking, alcohol use, physical activity, body mass index, and presence of heart disease, stroke, cancer and/or diabetes. The age- and sex-specific risks of a tobacco-attributable death were estimated by comparing the risk of death for smokers and non-smokers. The age- and sex-specific risks of a tobacco-attributable death (estimated in step 2 of the approach) were summed across the life course to estimate the lifetime risk of this measure. The age at which a tobacco-attributable death occurred was used to estimate the tobacco-attributable YLL. To properly scale the lifetime risk of a tobacco-attributable death and the associated YLL per cigarette smoked, age-specific risks for tobacco use were divided by the average number of cigarettes smoked by Canadians. The average number of cigarettes smoked by Canadians per day among smokers, by sex, was obtained from the 2017 Canadian Tobacco, Alcohol and Drugs Survey (24). This estimate relied on the assumption that the relative risk of death based on the number of cigarettes smoked per day is linear, and that this assumption is consistent with the observations from large cohort studies in the literature (25, 26). Details about the MPoRT model can be found in the Appendix.

### Exposure equivalency ratio

The exposure equivalency ratio for each alcohol use category was determined by dividing the tobacco equivalency, which is the ratio of the number of cigarettes smoked to produce a similar health loss or harm for a person’s alcohol use, by that person’s number of drinks/day.

### Uncertainty intervals

The 95% uncertainty intervals were based on a set of 1,000 simulations of all lowest level parameters (i.e., parameters sampled from their respective error distributions). These parameters were then utilized to estimate 1,000 simulated estimates. In these simulations, the 2.5th and 97.5th percentiles were the boundaries for the 95% uncertainty intervals (UIs).

## Results

**Figure 1:**
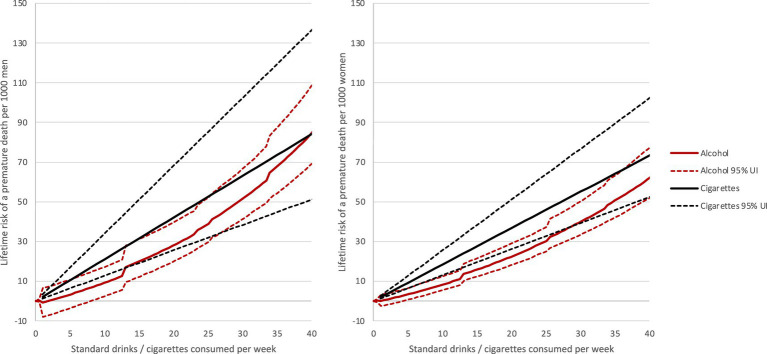
Lifetime deaths per 1,000 males and 1,000 females attributable to alcohol and tobacco use.

**Figure 2:**
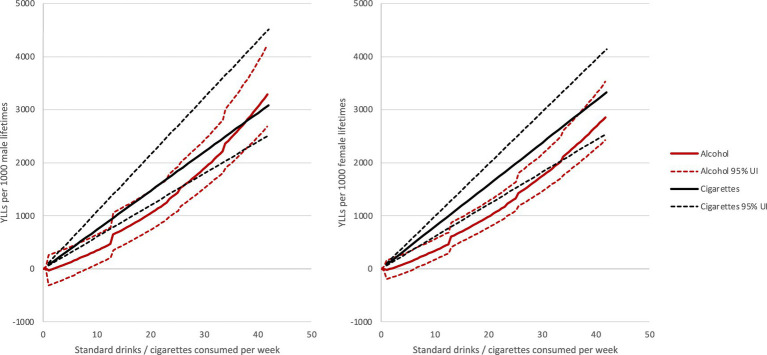
Years of life lost per 1,000 male and 1,000 female lifetimes attributable to alcohol and tobacco use.

As alcohol consumption increased, the equivalency ratio decreased for both males and females (Table 1; Figures 1, 2). For individuals who consumed 1 drink per day, each drink was equivalent to 0.4 cigarette smoked. For a male who consumes 5 drinks per day, the risk was equivalent to smoking 4.9 cigarettes per day. In other words, each drink was equivalent to one cigarette smoked. Similarly, for a female who consumes 5 drinks per day, the risk was equivalent to smoking 4.2 cigarettes per day (i.e., each drink was equivalent to 0.8 cigarette smoked). In all cases, evaluation of the risks were based on health loss or harm related to the two key study measures: premature death and YLL.

**Table 1:** Equivalency between alcohol consumed and cigarettes smoked based on attributable risks.

Sex	Drinks per day	(Grams of ethanol per week)*	Alcohol-attributable premature deaths per 1,000 lifetimes**		Alcohol-attributable YLL per 1,000 lifetimes**		Cigarette equivalency (per day)		Equivalence Ratio (cigarettes / standard drinks)	
							Based on deaths per 1,000 lifetimes	Based on YLL per 1,000 lifetimes		
									Deaths	YLL
Male										
	1	(98)	6.0	(−0.8, 13.7)	221.1	(−43, 514.9)	0.4	0.4	0.4	0.4
	2	(196)	19.4	(11.7, 30.5)	728.2	(429.4, 1142.5)	1.3	1.4	0.7	0.7
	3	(294)	31.7	(23.3, 43.5)	1170.8	(857, 1604.1)	2.2	2.3	0.7	0.8
	4	(392)	49.6	(39.7, 64.4)	1820.6	(1457.4, 2342.1)	3.4	3.5	0.8	0.9
	5	(490)	72.7	(58.7, 93.4)	2638.7	(2,135, 3,354)	4.9	5.1	1.0	1.0
Female										
	1	(98)	5.6	(2.9, 9.4)	219.6	(49.6, 430.7)	0.4	0.4	0.4	0.4
	2	(196)	15.4	(11.9, 21.0)	682.8	(491.3, 952.2)	1.2	1.2	0.6	0.6
	3	(294)	25.2	(20.6, 31.9)	1102.6	(884.1, 1404.4)	2.0	2.0	0.7	0.7
	4	(392)	38.6	(32.3, 48.6)	1687.1	(1410.8, 2092.4)	3.0	3.0	0.8	0.8
	5	(490)	54.0	(45.2, 67.4)	2346.9	(1972.4, 2889.6)	4.2	4.2	0.8	0.8

*Rounded to the nearest whole number. **95% uncertainty interval. PE, point estimate.

The equivalency ratio for alcohol use and tobacco use varied by sex. For females, consuming a standard drink did not result in as much of a negative impact on health (e.g., loss or harm) as smoking a cigarette; this was true for all alcohol use categories examined. For males who consume fewer than 5 drinks per day, drinking a standard drink also did not result in as much of a negative impact on health as smoking a cigarette. For males who consume 5 drinks per day, the risk associated with alcohol use was equal to that of tobacco use.

## Discussion

Risk equivalencies were explored to characterize the risks associated with alcohol use among Canadians as compared to risks associated with tobacco use, expressed in terms of the number of cigarettes smoked. For both males and females, a negative association was observed between alcohol use and equivalency ratios, such that as the number of standard drinks increased, equivalency ratios decreased. This result implies that for males who consume less than 5 drinks per day, consuming alcohol was less of a risk for health loss than smoking cigarettes, whereas for females, in all alcohol use categories examined, the risks associated with consuming alcohol were lower than the risks associated with smoking cigarettes. The fluctuation in these exposure equivalency ratios is likely related to the daily amount of alcohol consumed, as this amount is directly correlated with the risk of health loss or harm per unit of alcohol used (27). In contrast, the number of cigarettes smoked daily does not necessarily alter this relationship to the risk of health loss or harm per cigarettes smoked (25, 26). In other words, the number of standard drinks consumed per day carries a dose-dependent effect that the number of cigarettes smoked daily do not.

### Public health relevance

Given the magnitude of risks associated with alcohol use, and the generally poor communication of these risks to the target populations alcohol-related health outcomes (they are generally poor) when expressed as a measure of the number of cigarettes smoked, could resonate substantively better with the public than just straight statistics about alcohol consumption’s harm. Currently, the general public is not as aware of what constitutes a standard drink, let alone having adequate knowledge about the guidance on what is daily or weekly low-risk drinking; suffice to say, misconceptions about alcohol use are plenty (5, 28, 29). By contrast, tobacco-related risks are very well known to the general public, due in part to the heavy stigma associated with them and the decades of public health counter-advertising levied against the tobacco industry and their sales of tobacco products (30). As such awareness about risks associated with alcohol use could be enhanced if the public health or medical community uses similar public health messaging tactics and/or field tested strategies proven to work when communicating these risks. In a previous study, researchers showed that consumers of alcohol would decrease their drinking if they were informed about alcohol-attributable risks or harms — e.g., the link between excessive drinking and cancer (5). These consumers would take action (decrease drinking) if they were incentivized and understood alcohol-attributable consequences in more familiar terms. Since tobacco use is associated with several diseases that are also related to alcohol consumption (e.g., cancer, cardiovascular diseases, liver disease), it has become an example to emulate for communications purposes (31). However, a one-to-one translation of tobacco control strategies to reducing alcohol use may not be entirely possible since tobacco is highly addictive, the mechanisms of addiction may be different from alcohol, and most mainstream tobacco control interventions typically advocate for complete cessation (32). The approach to alcohol use, on the other hand, often relies on a harm reduction perspective, especially for those with problem drinking but not in full dependency. Furthermore, alcohol and tobacco use often occur concurrently. Thus, when conveying risk equivalencies to alcohol users it may be necessary to exercise caution in how risks are presented so as to avoid normalizing or downplaying the health consequences of tobacco use.

Risk perceptions about alcohol and tobacco use can also be influenced by the following factors: perceived benefit, immediacy of effect, knowledge about the risk to the exposed person, certainty of the scientific information regarding the risk, control over risk, newness of the risk, the severity of the consequences, and the extent to which each of these behaviors are normalized in society (33). These, factors are particularly important to consider when communicating risks about these two different behaviors. For example, the risk perception about alcohol use frequently is complicated by the fact that drinking alcohol has both protective and detrimental effects on health. Previous studies have noted that at low levels of consumption, alcohol use has a protective effect on ischemic heart disease, ischemic stroke, and diabetes; however, at the same level of consumption, there could also be a detrimental effect on other diseases, such as cancer (1). Thus, to tease out these more nuanced health impacts of alcohol use — i.e., protective versus detrimental — more objective measures of burden could be used to communicate accurate facts about alcohol’s effects, as has been done in this present study, which used premature death and YLL as primary measures to quantify potential health loss or harm (risks) associated with alcohol use.

Finally, it is also important to take into consideration the levels of alcohol versus tobacco use when communicating public health information. There is a notable difference in the number of people who consume alcohol versus those who use tobacco products. For example, in 2019, 76% of Canadian adults consumed alcohol while only 12% of adults smoked cigarettes in the past year (34, 35). This difference in prevalence between the two behaviors suggests a normalization of alcohol use versus tobacco use, likely the result of alcohol’s historical significance, coupled with its role in religious rituals, social gatherings, and cultural traditions. These various factors may have perpetuated the use of alcohol in spite of its known risks or harms, and inhibited public health’s progress to reform this behavior (36). In contrast, decades of public health policies and campaigns have rendered tobacco use more unacceptable and represent factors that have lowered smoking prevalence in Canada and elsewhere around the world (37).

### Limitations

The risk equivalency estimates reported here have numerous limitations that should be considered. First, the presented risk equivalencies do not take into account the following factors which may interact with alcohol and tobacco: harms to others, disability, non-health harms, and differences in an person’s risk factors . For example, both alcohol and tobacco carry risks for second-hand harms through non-user exposure—i.e., increased risk of alcohol-related injuries (e.g., motor vehicle accidents) or secondhand exposure due to drifting tobacco smoke (1, 38). Second-hand harms from alcohol and tobacco are both prevalent and difficult to avoid. They are dependent upon whether the exposure is acute or chronic. For instance second-hand harms from alcohol use are generally due to the intoxicating effects of the use (i.e., acute alcohol use) (1). Whereas second-hand harms through non-user exposure to tobacco smoke are generally not as acute, imparting a more cumulative effect over time (39). When communicating risks of these two substances, these indirect, second-hand harms, should be explained carefully, in conjunction with the more immediate, direct effects of their use.

Second, alcohol and tobacco have been noted to interact and share risk factors—e.g., and further interactions through co-use have been shown to exponentially increase the risk of head and neck cancers (1). Alcohol and tobacco use also tend to cluster with other chronic disease risks, such as high body mass index (BMI) (40). This clustering to other risks may be important to consider when formulating individualized treatment plans for patients with alcohol use disorders and tobacco dependence together. While some treatment programs may provide cigarettes as an incentive or reward in drug use treatment (41), this may be counterproductive (42). A systematic review found that in 16 out of 31 studies that examined pharmacological and psychotherapeutic alcohol use disorder treatments, being a non-smoker or having decreased tobacco consumption was significantly associated with reduced alcohol use (42). In stop-smoking studies, however, reduced smoking had no observable effect on drinking behaviors (42). In short, based on these results, treatment of alcohol use disorders may be more effective if tobacco addiction is also being treated concurrently.

Third and lastly, the data analysis and modeling employed in this study have several limitations. For example, the main analysis did not account for heavy drinking or patterns of drinking. Such indirect risks should be publicly communicated in addition to the direct harms experienced by users. Alcohol and tobacco use also contribute greatly to the risk of disability (43). Accordingly, the alcohol lifetime risk estimates are reflective of the patterns of drinking of the participants from the cohort and case–control studies that were used in the meta-analyses which reported cause-specific relative risk functions. There is evidence that drinking patterns affect the risk of infectious diseases (44), the risk of breast cancer (45), the risk of ischemic heart disease and ischemic stroke (46), the risk of diabetes (47), the risk of epilepsy (48), and the risk of injuries (49). Thus, if a person has a higher tendency to engage in heavy episodic drinking than the cohort and case–control participants, they would experience more health loss or harm than reported in the risk curves. If a person has a lower tendency to engage in heavy episodic drinking than the cohort and case–control participants, they will experience less health loss or harm than reported in the risk curves. The tendency to engage in heavy episodic drinking in the underlying cohort and case–control participants was not examined or reported in the meta-analyses.

As previously noted, the lifetime risk curves for alcohol were modeled for people who consume 0 to 5 standard drinks, these health loss or harms related to alcohol use would surpass those associated with tobacco use. It would be pertinent to explore this estimate, as it expands the utility of risk equivalency estimates in knowledge translation products—especially for people who are heavy chronic drinkers and people with alcohol use disorders.

The lifetime risk estimates for alcohol and tobacco use are derived using two different models, thereby limiting their comparability. Ideally, the risks of alcohol and tobacco use would be estimated using identical methods and data sources. The MPoRT model produces risk estimates for alcohol. These risk estimates are based on the reference group of non-drinkers and do not consider “sick quitters,” and therefore these risk estimates are biased and should not be used (17).

The data presented in this study are also based on population statistics for Canada. Therefore, the risks presented here apply to Canadians in general, but do not reflect the risks for specific Canadians. For example, for a person who consumes low amounts of alcohol, the risk of developing liver disease and dying from liver cirrhosis is highly dependent upon that person’s other risk factors, such as obesity and hepatitis infection (50). Canadians who are obese and/or have a hepatitis infection are at risk for death due to alcohol-attributable liver cirrhosis (i.e., a death that would not occur if the person abstained from alcohol); however, people without these co-occurring risk factors would not be at risk for death due to liver cirrhosis if they consumed small amounts of alcohol. Thus, it is important to note that the risk curves presented are for public health guidance, and are not meant to present health advice that is specific to a person.

The risk curves in this study only apply to people living in Canada. Risk curves for alcohol use were constructed using data from Canada, and are dependent upon the mortality risks of Canadians. Risk curves for tobacco use were based on the MPoRT model which is based on Canadian cohort data (23). Construction of risk curves for alcohol use is feasible for other countries around the world if there are data on alcohol use and mortality [see (14, 15)]. Similarly, tobacco risk curves could be constructed if there is country-specific cohort data or region-specific cohort data. Both of these endeavors may be arduous for low- and middle-income countries where such data are sparse (51, 52).

## Conclusion

The impacts of health risks from alcohol and tobacco use can be directly compared using standardized measurements of lifetime risk for premature death and YLL. While the equivalency estimates for alcohol and tobacco do not incorporate social harms, comparisons of alcohol to tobacco in standard units (number of drinks versus number of cigarettes) could provide alcohol users with a better understanding, in comparative terms, of the risks they partake when they drink.

## Data availability statement

Publicly available datasets were analyzed in this study. This data can be found at: 2022 Canadian Guidance on Alcohol and Health: https://www.ccsa.ca/sites/default/files/2022-08/CCSA-LRDG-Update-of-Canada%27s-LRDG-Final-report-for-public-consultation-en.pdf. MPoRT Model data: https://pubmed.ncbi.nlm.nih.gov/27529741/.

## Author contributions

HJ: Formal analysis, Investigation, Writing – original draft, Writing – review & editing. IS: Formal analysis, Investigation, Methodology, Writing – original draft, Writing – review & editing. JR: Conceptualization, Investigation, Writing – review & editing. SC: Formal analysis, Methodology, Writing – review & editing. AS: Formal analysis, Investigation, Methodology, Writing – review & editing. TS: Conceptualization, Investigation, Methodology, Writing – review & editing. CL: Conceptualization, Data curation, Investigation, Methodology, Writing – review & editing. NS: Data curation, Investigation, Methodology, Writing – review & editing. HE: Data curation, Investigation, Methodology, Writing – review & editing. PB: Data curation, Formal analysis, Funding acquisition, Investigation, Methodology, Project administration, Resources, Supervision, Visualization, Writing – original draft, Writing – review & editing. CP: Conceptualization, Data curation, Formal analysis, Investigation, Methodology, Project administration, Resources, Supervision, Validation, Visualization, Writing – original draft, Writing – review & editing. KS: Data curation, Formal analysis, Investigation, Methodology, Project administration, Supervision, Validation, Visualization, Writing – original draft, Writing – review & editing.
