# Supplementary material for: A drink equals how many cigarettes? Equating mortality risks from alcohol and tobacco use in Canada
Source: Front Public Health. 2024 Feb 27;12:1331190. doi: 10.3389/fpubh.2024.1331190 (PMC10928000; doi:10.3389/fpubh.2024.1331190)
Supplement: Supplementary file 1 [file Table_1.DOCX]

APPENDIX

A Drink Equals How Many Cigarettes? Equating Mortality Risks From Alcohol and Tobacco Use in Canada

**Lifetime risk of an alcohol-attributable death**

The details of how the number of alcohol-attributable deaths were estimated are outlined in the Canadian low-risk drinking guidelines report (see (1, 2)). These estimates are calculated by multiplying the risk of death for lifetime abstainers by age and sex and cause (see Table A1 for causes included) by corresponding cause-, sex- and age-specific relative risk (RR) estimates. The sources for the RR estimates are outlined in Table A2. These RRs used the reference group of people who engaged in lifetime abstention from alcohol and were corrected for people who engaged in past drinking (PD) (i.e., people who have consumed at least one standard drink of alcohol in their lifetime, but who have not consumed at least one standard drink of alcohol in the past year).

No latency period was used in the estimation of the number of alcohol-attributable deaths, except for deaths due to alcohol-attributable cancers. For cancer mortality and morbidity attributable to alcohol consumption, a latency period of 10 years was chosen between the consumption of alcohol and the diagnosis and/or death from cancer, based on an observed approximate latency period of 11 to 12 years for breast, colorectal, oral cavity, oesophageal (squamous cell carcinoma), and pharyngeal cancers, and 8 to 9 years for laryngeal and liver cancers (3).

A comparative risk assessment methodology was utilized to estimate the burden of disease among lifetime abstainers (by subtracting the number of alcohol-attributable deaths from the total number of deaths). For this method, only causes of death causally related to alcohol use, as assessed by their inclusion in the World Health Organization’s (WHO’s) Global Status Report on Alcohol and Health (GSRAH) (4) and/or the Institute for Health Mertrics and Evaluation’s Global Burden of Disease study (5), were included in the analysis (see Table A1).

We combined data on alcohol use, corresponding RR estimates (see Table A2) and mortality data to estimate the alcohol-attributable burden of disease. alcohol consumption statistics were obtained from 2009 for cancer (obtained from the Canadian Alcohol and Drug Use Monitoring Survey (CADUMS) 2009 (used as a proxy for 2007 to 2009) (6)) and from 2019 for all other diseases and injuries causally associated with alcohol use (obtained from the 2017 Canadian Tobacco, Alcohol and Drugs (CTADS) Survey (7) (used as a proxy for 2017 to 2019)). To correct for undercoverage in alcohol surveys, we also used adult *per capita* alcohol consumption data (see (8, 9)). For Canada, adult *per capita* alcohol consumption data were obtained for 2009 and 2019 from Statistics Canada (10), and unrecorded and tourist *per capita* alcohol consumption data were obtained from the WHO’s Global Information System on Alcohol and Health (11). A correction factor of 0.8 was applied to adult *per capita* alcohol consumption data to account for (i) alcohol that was not consumed, and (ii) the underreporting of alcohol consumption in medical observation studies from which the RR estimates used in this study were obtained (12).

**Table A1.** Alcohol-related diseases, conditions, and injuries

| **Cause categories** | | | | **ICD-10 codes** |
| --- | --- | --- | --- | --- |
| Communicable, maternal, perinatal and nutritional conditions | | | | A00-B99, D50-53, D64.9, E00-02, E40-46, E50-64, G00-04, G14, H65-66, J00-22, N70-73, O00-99, P00-96, U04 |
|  | Infectious and parasitic diseases | | | A00-B99, G00-04, G14, N70-73, P37.3, P37.4 |
|  |  | Tuberculosis | | A15-19, B90 |
|  | Respiratory infections | | | H65-66, J00-22, P23, U04 |
|  |  | Lower respiratory infections | | J09-22, P23, U04 |
| Noncommunicable diseases | | | | C00-97, D00-48, D55-64 (minus D64.9), D65-89, E03-07, E10–34, E65–88, F01–99, G06–98 (minus G14), H00–61, H68–93, I00–99, J30–98, K00–92, L00–98, M00–99, N00–64, N75–98, Q00–99, X41–42, X44, X45, R95 |
|  | Malignant neoplasms | | | C00–97 |
|  |  | Mouth and oropharynx cancers | | C00–14 |
|  |  |  | Lip and oral cavity | C00–08 |
|  |  |  | other pharyngeal cancers | C09–10, C12–14 |
|  |  | Oesophagus cancer | | C15 |
|  |  | Colon and rectum cancers | | C18–21 |
|  |  | Liver cancer | | C22 |
|  |  | Breast cancer | | C50 |
|  |  | Larynx cancer | | C32 |
|  | Diabetes mellitus | | | E10–14 (minus E10.2–10.29, E11.2–11.29, E12.2, E13.2–13.29, E14.2) |
|  | Mental and substance use disorders | | | F04–99, G72.1, Q86.0, X41–42, X44, X45 |
|  |  | Alcohol use disorders | | F10, G72.1, Q86.0, X45 |
|  | Neurological conditions | | | F01–03, G06–98 (minus G14, G72.1) |
|  |  | Degeneration of nervous system due to alcohol | | G31.2 |
|  |  | Epilepsy | | G40–41 |
|  |  | Alcohol polyneuropathy | | G62.1 |
|  |  | Alcoholic myopathy | | G72.1 |
|  | Cardiovascular diseases | | | I00–99 |
|  |  | Hypertensive heart disease | | I10–15 |
|  |  | Ischaemic heart disease | | I20–25 |
|  |  | Stroke | | I60–69 |
|  |  |  | Ischaemic stroke | G45–46.8, I63–63.9, I65–66.9, I67.2–67.848, I69.3–69.4 |
|  |  |  | Intracerebral haemorrhage | I61-I62, I62.9, I69.0-I69.298 |
|  |  |  | Subarachnoid hemorrhage | I60-I60.9, I67.0-I67.1 |
|  |  | Cardiomyopathy, myocarditis, endocarditis | | I30–33, I38, I40, I42 |
|  |  |  | Alcohol cardiomyopathy | I42.6 |
|  |  | Atrial fibrillation and flutter | | I48 |
|  | Digestive diseases | | | K20–92 |
|  |  | Alcoholic gastritis | | K29.2 |
|  |  | Cirrhosis of the liver | | K70, K74 |
|  |  | Pancreatitis | | K85–86 |
|  |  | Fetus and newborn affected by maternal use of alcohol | | P04.3 |
| Injuries | | | | V01–Y89 (minus X41–42, X44, X45) |
|  | Unintentional injuries | | | V01–X40, X43, X46–59, Y40–86, Y88, Y89 |
|  |  | Road injury | | V01–04, V06, V09–80, V87, V89, V99* |
|  |  | Poisonings | | X40, X43, X46–48, X49 |
|  |  | Falls | | W00–19 |
|  |  | Fire, heat and hot substances | | X00–19 |
|  |  | Drowning | | W65–74 |
|  |  | Exposure to mechanical forces | | W20–38, W40–43, W45, W46, W49–52, W75, W76 |
|  |  | Other unintentional injuries | | Rest of V, W39, W44, W53–64, W77–99, X20–29, X50–59, Y40–86, Y88, Y89 |
|  | Intentional injuries | | | X60–Y09, Y35–36, Y870, Y871 |
|  |  | Self-harm | | X60–84, Y870 |
|  |  | Interpersonal violence | | X85–Y09, Y871 |

**Table A2.** Relative risk estimates by disease category

| **Cause categories** | | | | **RR source*** |
| --- | --- | --- | --- | --- |
| Communicable, maternal, perinatal and nutritional conditions | | | |  |
|  | Infectious and parasitic diseases | | |  |
|  |  | Tuberculosis | | (13) |
|  | Respiratory infections | | |  |
|  |  | Lower respiratory infections | | (14) |
| Noncommunicable diseases | | | |  |
|  | Malignant neoplasms | | |  |
|  |  | Mouth and oropharynx cancers | |  |
|  |  |  | Lip and oral cavity | (15) |
|  |  |  | other pharyngeal cancers | (15) |
|  |  | Oesophagus cancer | | (15) |
|  |  | Colon and rectum cancers | | (16) |
|  |  | Liver cancer | | (17) |
|  |  | Breast cancer | | (18) |
|  |  | Larynx cancer | | (15) |
|  | Diabetes mellitus | | | (19) |
|  | Mental and substance use disorders | | |  |
|  |  | Alcohol use disorders | | 100% alcohol attributable |
|  | Neurological conditions | | |  |
|  |  | Degeneration of nervous system due to alcohol | | 100% alcohol attributable |
|  |  | Epilepsy | | (20) |
|  |  | Alcohol polyneuropathy | | 100% alcohol attributable |
|  |  | Alcoholic myopathy | | 100% alcohol attributable |
|  | Cardiovascular diseases | | |  |
|  |  | Hypertensive heart disease | | (21) |
|  |  | Ischaemic heart disease | | (22) |
|  |  | Stroke | |  |
|  |  |  | Ischaemic stroke | (23) |
|  |  |  | Intracerebral haemorrhage | (23) |
|  |  |  | Subarachnoid hemorrhage | (23) |
|  |  | Cardiomyopathy, myocarditis, endocarditis | |  |
|  |  |  | Alcohol cardiomyopathy | 100% alcohol attributable |
|  |  | Atrial fibrillation and flutter | | (24) |
|  | Digestive diseases | | |  |
|  |  | Alcoholic gastritis | | 100% alcohol attributable |
|  |  | Cirrhosis of the liver | | (25) |
|  |  | Pancreatitis | | (26) |
|  |  | Fetus and newborn affected by maternal use of alcohol | | 100% alcohol attributable |
| Injuries | | | |  |
|  | Unintentional injuries | | |  |
|  |  | Road injury | | Shape of the RR curve: (27); Area under the RR curve: (28) |
|  |  | Poisonings | | Shape of the RR curve: (29); Area under the curve: (30) |
|  |  | Falls | | Shape of the RR curve: (29); Area under the curve: (30) |
|  |  | Fire, heat and hot substances | | Shape of the RR curve: (29); Area under the curve: (30) |
|  |  | Drowning | | Shape of the RR curve: (29); Area under the curve: (30) |
|  |  | Exposure to mechanical forces | | Shape of the RR curve: (29); Area under the curve: (30) |
|  |  | Other unintentional injuries | | Shape of the RR curve: (29); Area under the curve: (30) |
|  | Intentional injuries | | |  |
|  |  | Self-harm | | Shape of the RR curve: (29); Area under the curve: (30) |
|  |  | Interpersonal violence | | Shape of the RR curve: (29); Area under the curve: (30) |

The increased risk of death and disability for causes which are 100% attributable to alcohol was not modelled (see section D.1 for further details).

**Lifetime risk of a smoking-attributable death**

The Mortality Population Risk Tool (MPoRT) model was used to estimate the number of smoking-attributable premature deaths (i.e., deaths that occur before 75 years of age), and the number of years of life lost (YLL) attributable to smoking. The MPoRT model estimates the yearly probability of death based on age, smoking status (light, heavy or former), diet score, alcohol use, neighbourhood deprivation, education, body mass index, years since immigration, and having heart disease, a previous stroke, cancer, or diabetes. Beta coefficients and reference values for the MPoRT model were taken from the MPoRT model by Manuel and colleagues (except for smoking status which was modified to assess the smoking-attributable deaths) (31). For the estimations we used a lifetime approach, and assumed that at 20 years of age (the start year of the MPoRT model) no one in the model was an immigrant to Canada, and no one had heart disease, a previous stroke, cancer or diabetes (the risk of heart disease, stroke and cancer are all affected by smoking and therefore the effect of smoking on mortality may be mediated through these variables).

The one-year probability of death was estimated using Formula A1. The values for h_Adj_ were taken from the paper from Manuel and colleagues (i.e., 3.72903E-05 for males and 2.38108E-05 for females) (31). Values for h_Cal_ were obtained by sex and age and were estimated based on the difference in the observed and predicted risk in the study by Manuel and colleagues (31). The Score(t) was estimated based on the MPoRT model by Manuel and colleagues, based on age (represented by a), smoking status (light, heavy or former (represented by c)), diet score, alcohol use, neighbourhood deprivation, education, body mass index, years since immigration, and having heart disease, a previous stroke, cancer, or diabetes. The t score was estimated by sex (represented by s).

*Formula A1*

$${P\left( 1 Year \right)}_{a,s,c}= 1- {(h}_{Adj}\cdot exp\left[ Score\left( t_{a,s,c} \right) \right]\cdot h_{Cal; a,s})$$

The smoking-attributable probability of death (represented by AP) was estimated for light smokers and heavy smokers by subtracting the P(1 year) for light and heavy smokers from the P(1 year) for non-smokers (see Formula A2). The number of people alive for each year was estimated using a lifetime approach and using one-year increments from 20 to 99 years of age. The number of smokers, heavy smokers, and light smokers alive at the end of each year of life was estimated by multiplying the probability of survival for a given age by the number of people alive at the beginning of each year of life (see Formula A3).

*Formula A2*

$${AP\left( 1 Year \right)}_{a,LS/HS,c}={P\left( 1 Year \right)}_{a,NS,c}-{P\left( 1 Year \right)}_{a,LS/HS,c}$$

*Formula A3*

$${Alive}_{a,s,c}= {Alive}_{a-1,s,c}\cdot{P\left( 1 Year \right)}_{a,s,c}$$

The smoking-attributable deaths were then estimated by multiplying the smoking-attributable probability of death by the number of light smokers and heavy smokers by age (see Formulas A4 and A5). The smoking-attributable YLL were then estimated by multiplying the smoking-attributable deaths by age and sex with the corresponding number of YLL.

**References**

1. Shield K. D., Gmel G., Gmel G., Mäkelä P., Probst C., Room R. et al. Life‐time risk of mortality due to different levels of alcohol consumption in seven European countries: implications for low‐risk drinking guidelines, Addiction 2017: 112: 1535-1544.

2. Shield K., Churchill S., Sherk A., Stockwell T., Lévesque C., Sanger N. et al. Lifetime Risk of Alcohol-Attributable Death and Disability, Ottawa, Canada: Canadian Centre on Substance Use and Addiction In Press.

3. Grundy A., Poirier A. E., Khandwala F., McFadden A., Friedenreich C. M., Brenner D. R. Cancer incidence attributable to alcohol consumption in Alberta in 2012, CMAJ open 2016: 4: E507.

4. World Health Organization. Global status report on alcohol and health 2018, Geneva: World Health Organization; 2018.

5. Institute of Health Metrics and Evaluation. GBD Results Tool, Seattle, USA: Institute of Health Metrics and Evaluation; 2021.

6. Health Canada. Canada Alcohol and Drug Use Monitoring Survey 2009., Ottawa, Canada: Health Canada; 2010.

7. Government of Canada. Canadian Tobacco Alcohol and Drugs (CTADS) Survey: 2017 summary, Ottawa, Canada; 2018.

8. Kehoe T., Gmel G., Shield K. D., Gmel G., Rehm J. Determining the best population-level alcohol consumption model and its impact on estimates of alcohol-attributable harms, Population Health Metrics 2012: 10: 6.

9. Rehm J., Kehoe T., Gmel G., Stinson F., Grant B., Gmel G. Statistical modeling of volume of alcohol exposure for epidemiological studies of population health: the US example, Population Health Metrics 2010: 8: 1-12.

10. Statistics Canada. Table 10-10-0010-01 Sales of alcoholic beverages types by liquor authorities and other retail aoutlets, by value, volume, and absolute volume: Statistics Canada; 2021.

11. World Health Organization. Global Information System on Alcohol and Health, Geneva, Switzerland: World Health Organization; 2021.

12. Gmel G., Rehm J. Measuring alcohol consumption, Contemporary Drug Problems 2004: 31: 467.

13. Imtiaz S., Shield K. D., Roerecke M., Samokhvalov A. V., Lönnroth K., Rehm J. Alcohol consumption as a risk factor for tuberculosis: meta-analyses and burden of disease, Eur Respir J 2017: 50: 1700216.

14. Samokhvalov A., Irving H., Rehm J. Alcohol consumption as a risk factor for pneumonia: a systematic review and meta-analysis, Epidemiol Infect 2010: 138: 1789-1795.

15. Bagnardi V., Rota M., Botteri E., Tramacere I., Islami F., Fedirko V. et al. Alcohol consumption and site-specific cancer risk: a comprehensive dose-response meta-analysis, Br J Cancer 2015: 112: 580-593.

16. Vieira A., Abar L., Chan D., Vingeliene S., Polemiti E., Stevens C. et al. Foods and beverages and colorectal cancer risk: a systematic review and meta-analysis of cohort studies, an update of the evidence of the WCRF-AICR Continuous Update Project, Annals of Oncology 2017: 28: 1788-1802.

17. World Cancer Research Fund/American Institute for Cancer Research. Diet, nutrition, physical activity and cancer: a global perspective. Continuous Update Project expert report 2018. , London, United Kingdom; 2018.

18. Sun Q., Xie W., Wang Y., Chong F., Song M., Li T. et al. Alcohol consumption by beverage type and risk of breast cancer: a dose-response meta-analysis of prospective cohort studies, Alcohol and Alcoholism 2020: 55: 246-253.

19. Knott C., Bell S., Britton A. Alcohol consumption and the risk of type 2 diabetes: a systematic review and dose-response meta-analysis of more than 1.9 million individuals from 38 observational studies, Diabetes Care 2015: 38: 1804-1812.

20. Samokhvalov A. V., Irving H., Mohapatra S., Rehm J. Alcohol consumption, unprovoked seizures, and epilepsy: A systematic review and meta‐analysis, Epilepsia 2010: 51: 1177-1184.

21. Liu F., Liu Y., Sun X., Yin Z., Li H., Deng K. et al. Race-and sex-specific association between alcohol consumption and hypertension in 22 cohort studies: A systematic review and meta-analysis, Nutrition, Metabolism and Cardiovascular Diseases 2020: 30: 1249-1259.

22. Zhao J., Stockwell T., Roemer A., Naimi T., Chikritzhs T. Alcohol consumption and mortality from coronary heart disease: an updated meta-analysis of cohort studies, Journal of studies on alcohol and drugs 2017: 78: 375-386.

23. Larsson S. C., Wallin A., Wolk A., Markus H. S. Differing association of alcohol consumption with different stroke types: a systematic review and meta-analysis, BMC medicine 2016: 14: 1-11.

24. Larsson S. C., Drca N., Wolk A. Alcohol consumption and risk of atrial fibrillation: a prospective study and dose-response meta-analysis, Journal of the American College of Cardiology 2014: 64: 281-289.

25. Roerecke M., Vafaei A., Hasan O. S. M., Chrystoja B. R., Cruz M., Lee R. et al. Alcohol consumption and risk of liver cirrhosis: a systematic review and meta-analysis, Am J Gastroenterol 2019: 114: 1574-1586.

26. Samokhvalov A. V., Rehm J., Roerecke M. Alcohol consumption as a risk factor for acute and chronic pancreatitis: a systematic review and a series of meta-analyses, EBioMedicine 2015: 2: 1996-2002.

27. Taylor B., Rehm J. The relationship between alcohol consumption and fatal motor vehicle injury: high risk at low alcohol levels, Alcoholism: Clinical and Experimental Research 2012: 36: 1827-1834.

28. Brown S. W., Vanlaar W. G. M., Robertson R. D., The Traffic Injury Research Foundation of Canada. The Alcohol and Drug Crash Problem in Canada 2016 Report, Ottawa, Canada: Canadian Council of Motor Transport Administrators; 2021.

29. Taylor B., Irving H., Kanteres F., Room R., Borges G., Cherpitel C. et al. The more you drink, the harder you fall: a systematic review and meta-analysis of how acute alcohol consumption and injury or collision risk increase together, Drug and Alcohol Dependence 2010: 110: 108-116.

30. Canadian Institute for Health Information. National Trauma Registry 2009 Report: Major Injury in Canada, Ottawa, Canada; 2010.

31. Manuel D. G., Perez R., Sanmartin C., Taljaard M., Hennessy D., Wilson K. et al. Measuring burden of unhealthy behaviours using a multivariable predictive approach: life expectancy lost in Canada attributable to smoking, alcohol, physical inactivity, and diet, PLoS medicine 2016: 13: e1002082.
